# Pediatric COVID-19 Therapeutics: Seizing the Right Research and Development Opportunities to Accelerate Access for Children

**DOI:** 10.1097/INF.0000000000003331

**Published:** 2021-11-26

**Authors:** Sébastien Morin, Marc Lallemant, Anthony Garcia-Prats, Linda Lewis, Melynda Watkins, Carlo Giaquinto, Marie Valentin, Martina Penazzato, John C. Reeder

**Affiliations:** From the * Medicines Patent Pool, Geneva, Switzerland; † Faculty of Associated Medical Sciences, Chiang Mai University, Chiang Mai, Thailand; ‡ Department of Pediatrics, University of Wisconsin School of Medicine and Public Health, Madison, Wisconsin, USA; § Desmond Tutu TB Centre, Department of Paediatrics and Child Health, Faculty of Medicine and Health Sciences, Stellenbosch University, Cape Town, South Africa; ¶ Clinton Health Access Initiative, Boston, Massachusetts, USA; ‖ Department of Women and Child Health, University of Padova, Padova, Italy; ** World Health Organization, Geneva, Switzerland.

**Keywords:** Pediatrics, COVID-19, SARS-CoV-2, therapeutics, research and development

## Abstract

Children, although at lower risk of poor outcomes from COVID-19 relative to adults, still stand to benefit from therapeutic interventions. Understanding of COVID-19 clinical presentation and prognosis in children is essential to optimize therapeutic trials design. This perspective illustrates how to collectively accelerate pediatric COVID-19 therapeutic research and development, based on the experience of the Global Accelerator for Paediatric Formulations.

The development of pediatric medicines is often an afterthought, and COVID-19 is no exception. While research and development of therapeutics proceeds at unprecedented speed, children remain neglected. At lower risk of severe COVID-19, children are important in the transmission dynamic^1^ and still stand to benefit personally from safe, effective COVID-19 treatments. Small but meaningful proportions of children require intensive care and children with chronic conditions might be at higher risk for severe disease and death.^2–4^ This is especially true in low- and middle-income countries (LMICs) where optimized supportive measures are not consistently available.^2^ Additionally, treatments to reduce viral shedding might play an important role in breaking the chain of transmission, especially as more transmissible variants spread in the population and more concerns are raised for post-COVID conditions. Therefore, timely and appropriate consideration of children in the development of COVID-19 therapeutics must be ensured.

The Global Accelerator for Paediatric Formulations (GAP-f, https://www.who.int/initiatives/gap-f), a World Health Organization (WHO) Network, aims to stimulate cross-sectoral collaborations to accelerate investigation, development, registration, and uptake of prioritized child-appropriate medicines.^5^ Here we provide a perspective from GAP-f on how the international community can collaboratively accelerate research, development, and access to COVID-19 therapeutics for children. Although the development of pediatric COVID-19 vaccines is urgent too, this warrants separate considerations to therapeutics, and vaccines will not be discussed further.

## Evolving Understanding of COVID-19 in Children and Implications for Pharmacologic Treatments

The understanding of the epidemiology, clinical presentation and prognosis of COVID-19 in children as compared with adults is still emerging, and systematic studies, such as the “Human Epidemiology and Response to SARS-CoV-2” study,^6^ are critical for informing how pediatric trials should be designed.^7^ When disease course and response to the medicinal product are similar in adults and children, regulatory agencies such as the United States Food and Drug Administration and the European Medicines Agency support extrapolating efficacy results from adult trials to children. This reduces the complexity and time required for registering new therapeutics for children, and highlights the need for careful characterization of the disease in adults and children.^8,9^ Pharmaceutical companies are encouraged to discuss with regulators early about their pediatric clinical development plans, including extrapolation of efficacy data from adult studies, pediatric pharmacokinetic (PK) trials to support dose selection and size of preapproval pediatric safety databases.^8^

Determining whether extrapolation is appropriate for different COVID-19 therapeutics is a central challenge for developing treatments for children. While the initial course of SARS-CoV-2 infection in children is similar to adults,^10^ progression to severe disease is much less frequent, with infants (<1 year of age), and children and adolescents with chronic conditions having a higher risk.^3,11^ In addition,^12^ emerging evidence suggests that children may play a major role in household and community transmission.^13,14^ Therefore, research to better characterize the role of children in onward transmission will help assess the potential value of therapeutics to reduce infectivity.

In the later stages of disease in adults, the more severe manifestations are associated with a dysregulated, often excessive, immune response triggered by the virus.^15^ The acute respiratory distress syndrome characteristic of severe COVID-19 in adults is less frequently observed in adolescents, and only rarely in young children.^16^ Conversely, an equivalent for the children and adolescents multisystem inflammatory syndrome (MIS-C) with elevated inflammatory markers, multiorgan dysfunction and shock has not yet been identified in adults.^17,18^ In these instances, extrapolation from adult trials is an unlikely possibility.^19^

## Current Research Landscape of COVID-19 Pharmacologic Treatments

Although they may appear slow in a crisis, well-designed randomized comparative clinical trials are necessary to rigorously establish the safety and efficacy of therapeutic candidates. Among collaborative research and development efforts to address the pandemic, large international adaptative trials, including SOLIDARITY,^20^ ACTT,^21^ DISCOVERY^22^ and RECOVERY,^23^ evaluate multiple treatments simultaneously in diverse populations. Although complex, such trials are ideally suited to the urgency and uncertainty of the pandemic, as they allow design adaptation based on evolving data over time, including evolution of the standard of care, and addition or removal of tested products for futility or proven efficacy.^24^ While the Access to COVID-19 Tools Accelerator (ACT-A) partnership launched by WHO and partners, involving governments, scientists, civil society, funding agencies and international health organizations to accelerate the development, production and equitable distribution of COVID-19-related health products, including therapeutics,^25^ “aim of equitable global access to innovative tools for COVID-19 for all” should ensure appropriate inclusion of children in the development of medicines,^26^ the trials listed above, and others, have so far mostly targeted adults.

Whilst SOLIDARITY, ACTT, DISCOVERY and PRINCIPLE restricted enrollment to adults, RECOVERY included children. More broadly, a search as of June 20, 2021 of the ClinicalTrials.gov website found 1687 phase 1-3 COVID-19 drug trials, 96 (6%) of which are open to enrollment for children (43% planning to recruit up to 100 participants, see Table 1). Only 7 (7%) of these trials were specifically designed for children (ie, are enrolling children only). Most trials targeted severe disease and evaluated a very broad variety of products. Although inclusion of children seems valuable, the extreme heterogeneity of these trials in terms of products, design, inclusion criteria, clinical endpoints and sample sizes calls for caution.^27,28^ While providing children access to otherwise unavailable drugs, many trials appear unlikely to yield sufficient data to draw reasonable conclusions about their pharmacokinetics, dosing or safety putting children at risk of toxicities for little discernible benefits for them and the community at large.^29^

**Table 1.** Characteristics of COVID-19 Trials That Include Children Identified in ClinicalTrials.gov

A. Summary	
COVID-19 phase 1–3 trials identified	1687
Trials allowing inclusion of children	96 (6%)*
B. Pediatric populations in trials allowing inclusion of children	
Adult trials allowing inclusion of children	89 (93%)
Eligibility at unspecified age	31 (35%)
Eligibility below 1 y of age	2 (2%)
Eligibility between 1 and 10 y of age	10 (11%)
Eligibility between 10 and 14 y of age	19 (21%)
Eligibility above 15 y of age	27 (30%)
Pediatric trials (only children included)	7 (7%)
C. Products being evaluated†	162
Products with anticipated antiviral activity	94 (58%)
New anti-infectives	36 (38%)
Remdesivir	10 (11%)
Chloroquine, hydroxychloroquine	11 (12%)
Monoclonal antibodies	15 (16%)
Repurposed anti-infectives	46 (49%)
Antibiotics	9 (10%)
Antiparasitic drugs	15 (16%)
Antiretrovirals	6 (6%)
Antivirals	16 (17%)
Herbal medicines and vitamin supplements	12 (13%)
Products with anticipated anti-inflammatory and/or anti-ARDS activity	60 (37%)
Monoclonal antibodies	12 (20%)
Corticosteroids	6 (10%)
Other anti-inflammatory products	12 (20%)
T-cell or stem cell infusions	8 (13%)
Convalescent plasma/immunoglobulins	19 (32%)
Anticoagulants	3 (5%)
Products with anticipated prevention utility	8 (5%)
Vitamin D, A or B	5, 2, 1 (63%, 25%, 13%)

Data as of 20 June 2021.

*Among the 96 trials open to enrollment for children, 43% are planning to recruit up to 100 participants only.

†Twenty-nine (30%) of the trials listed evaluated >1 product.

ARDS, acute respiratory distress syndrome.

In addition, while children should not be exposed to unnecessary risk from products that have not shown indication of safety and benefit in adult studies, deferring pediatric medicines evaluation until after completion of late-stage adult trials may delay access to effective treatments and increase children exposure to experimental use of medicines outside clinical trials.^30^ Given the political and societal pressure for expeditiously rolling out new drugs before supporting evidence in target populations is available, real-world data collection to monitor effectiveness and safety may be a complementary component of drug evaluation. However, recent reports of 2 large observational studies specifically targeting pediatric MIS-C,^31,32^ have provided conflicting findings regarding the efficacy of intravenous immunoglobulins, glucocorticoids or both, highlighting again the critical role of well-designed randomized trials in defining optimal therapeutic approaches. In some countries,^33^ monoclonal antibodies targeting the SARS-CoV-2 spike protein have been approved for early disease stage^34^ in mildly symptomatic children above 12 years of age and with underlying conditions (eg, cancer, immunodeficiency, uncontrolled diabetes or asthma). It is urgent to collect data to ensure appropriate, safe and effective use of such products in children, and trials enrolling children should ensure that relevant, actionable pediatric outcomes—pharmacokinetics, dosing, and safety—can be achieved through their inclusion.

## Critical Actions to Accelerate Access to COVID-19 Treatments for Children

GAP-f has developed a framework for accelerating access to pediatric formulations across the entire cycle of drug development; this includes: (1) prioritization and evaluation; (2) development and registration; and (3) delivery and safe use (see Table 2).^5^ Addressing bottlenecks at each step has sped children access to antiretrovirals and other anti-infectives, and application of the GAP-f framework to pediatric COVID-19 treatments is highly relevant (as detailed below and in Table 2).

**Table 2.** Priority Actions to Accelerate Access to COVID-19 Pharmacologic Treatments for Children

1) Prioritization and evaluation: Develop a clear prioritized drug portfolio, ensure completion of actionable pediatric clinical trials and facilitate regulatory submission and approvals
• Continue ongoing studies to better characterize the epidemiology, pathophysiology, clinical presentation, infectivity and outcomes of COVID-19 in children, to inform decisions about extrapolation from adult data and optimal pediatric trial design
• Avoid enrolling children in small, underpowered clinical trials and/or of insufficiently promising drug candidates
• Prospectively involve pediatricians in the design, implementation and monitoring of trials in large adaptive trial platforms, even when those are initially restricted to adults
• Rapidly evaluate PK, dose, safety and tolerability in children of therapeutics that have already demonstrated efficacy and safety in adults, extrapolating adult efficacy results to children when appropriate
• Include adolescents in adult trials when appropriate
• Proactively support trial sponsors of new drug candidates to develop pediatric investigation/study plans (PIPs and PSPs), and engage the pediatric scientific community (eg, GAP-f and other pediatric research networks) around those plans early on
2) Development and registration: Establish, support, maintain, and coordinate product development efforts
• Ensure rapid and coordinated development of age-appropriate formulations of any treatments through collaboration between industry, academic institutions, product development partnerships and key pediatric networks (such as GAP-f)
• Consider stability, storage, and packaging requirements for distribution and use in LMICs early on
• Accelerate regulatory review processes through regulatory cooperation and reliance approaches
3) Delivery and safe use: Ensure products are introduced rapidly in a coordinated manner, and healthcare systems and workers are prepared
• Develop access plans that specifically consider introduction and roll-out of pediatric formulations for LMICs as soon as new products are expected and early in the development process (this may involve generic manufacturers and access-oriented public-health voluntary licensing agreements through MPP)
• Align clinical guidance between WHO, national authorities, and medical associations
• Coordinate procurement logistics among various agencies, both internationally (such as with the Global Fund, PAHO Strategic Fund, UNDP and UNICEF) and nationally

Based on the GAP-f framework.

MPP, Medicines Patent Pool.

### Prioritization and Evaluation

Pediatric clinical research for COVID-19 therapeutics should focus on the most promising candidates, with early and clearly defined target product profiles. Without prioritization, fragmentation of clinical research efforts undermines the timely production of conclusive evidence (see Table 1). WHO-led prioritization processes for HIV,^35^ HCV and tuberculosis^36^ provide examples of focused efforts to achieve impact. While a target product profile for COVID-19 therapeutics has been developed by WHO acknowledging the importance of addresssing the needs of children, prioritization of COVID-19 therapeutics for pediatric investigation and development has not yet been done but is of critical importance, and leadership in this area would represent a substantial contribution.

To reduce the gap between development of medicines for adults and children, pediatric studies must build on existing best practices, using PK modeling and simulations to achieve drug exposure targets associated with efficacy and safety, and weight bands rather than age to define pediatric cohorts.^37^ Large, standardized cohorts simultaneously recruiting across all pediatric groups are needed to better understand COVID-19 pathophysiology, clinical and biologic evolution in adults and children. The WHO’s *Global COVID-19 Clinical Data Platform for clinical characterization and management of hospitalized patients with suspected or confirmed COVID-19*^38^ and the Coronavirus Clinical Characterization Consortium^39^ standardized data collection and analysis tools with clear diagnostic and inclusion criteria, cohorts characterization and harmonized endpoints, supports productive data sharing and meta-analyses. These initiatives are essential to determine which children are likely to benefit from therapeutics, allow their appropriate inclusion in clinical trials, and inform when extrapolation from adult studies may be sufficient to characterize efficacy in children.

Including adolescents who have similar disease progression and usually receive similar drug doses than adults in adult trials is supported by the FDA and should systematically be considered.^8,9^

Conversely, the inclusion of small numbers of children in all adult trials to acquire pediatric PK data for medicines with unproven efficacy is not an end in itself: children should not participate in trials that may potentially compromise their safety without clearly foreseeable benefits. A priority is the rapid pediatric evaluation of therapeutics that have already demonstrated efficacy and safety in adults. Existing and newly established large international adaptive or platform trials such as those mentioned above should include a procedure to enroll children in specifically designed pediatric subprotocols confirming PK in addition to characterizing tolerability and side effects. Multidisciplinary pediatric teams involved from the outset in trials recruiting adults could quickly activate the pediatric component when the first results show an early favorable risk-benefit balance for the most promising products. When applicable, population PK modeling using sparse sampling would provide essential data to guide dosing in children. International coordination is critical for coordinating sites, harmonizing endpoints and making optimal use of the relatively limited pediatric patient pools.

### Development and Registration

It is critical to ensure that future therapeutic options for children (particularly outside of hospital settings) are age-appropriate and adapted for use in LMICs. Originator and generic pharmaceutical partners, academic institutions and product development partnerships should collaborate with key networks, such as GAP-f, to define optimal target product profiles, and address barriers to developing and registering these formulations.

Regulatory review processes often remain fragmented, and reliance approaches to facilitate registration should be used, like the Collaborative Registration Procedure between the WHO Prequalification of Medicines Programme and National Regulatory Authorities^40^ and the Stringent Regulatory Authorities Collaborative Registration Procedure.^41^

Securing and sustaining manufacturing capacity of quality-assured affordable supplies to LMICs requires partnerships with both originator and generic manufacturers. Critical to this is clinical data sharing, technology transfers (especially in the case of complex manufacturing requirements) and access-oriented public-health voluntary licensing agreements, as with the UN-backed Medicines Patent Pool.^42^

### Delivery and Safe Use

Broad introduction and roll-out of new drug formulations also requires coordinated international and national efforts. Downstream activities include adoption in international and national clinical guidelines, procurement through international and domestic mechanisms, roll-out by governments and other implementing partners and treatment uptake by target populations. Access plans, especially for LMICs where fragile health care systems may be severely impacted by COVID-19, must be developed as soon as new products are expected. Coordination of procurement should ideally rely on international procurement agencies with existing footprints, such as the Global Fund, PAHO Strategic Fund, UNDP and UNICEF. Aligned clinical guidance between WHO,^43^ national authorities and medical associations, and dissemination of information to healthcare providers and affected communities should facilitate uptake.

## CONCLUSION

While the COVID-19 burden is concentrated in adults, children are also affected and deserve to benefit from advances in therapeutics, particularly considering their potential for onward transmission and emerging variants that bring uncertainty about epidemiology and clinical presentation of COVID-19 in children. Experience has shown that without intentional and coordinated action, children will be forgotten in the search for new treatments. COVID-19 represents an opportunity to re-envision how research and development can be undertaken and how political will, partnerships, and effective collaborations can be truly transformational.

## ACKNOWLEDGMENTS

The authors thank Janet V. Diaz, lead of clinical management in WHE at WHO, as well as members and partners of the Global Accelerator for Paediatric Formulations (GAP-f, https://www.who.int/initiatives/gap-f).
